# Supplementary material for: Multipurpose Processing Additives for Silica/Rubber Composites: Synthesis, Characterization, and Application
Source: Polymers (Basel). 2021 Oct 20;13(21):3608. doi: 10.3390/polym13213608 (PMC8587921; doi:10.3390/polym13213608)
Supplement: Supplementary file 1 [file polymers-13-03608-s001.zip › polymers-1402659-supplementary.pdf]

# Supporting Information

## Multipurpose processing additives for silica/rubber composites: synthesis, characterization, and application

**Arpan Datta Sarma[1, 2], Carlos Eloy Federico[1], Frida Nzulu[3], Marc Weydert[3], Pierre Verge[1], Daniel Frederick Schmidt[1,*]**

[1] Luxembourg Institute of Science and Technology, Department of Materials Research and Technology (MRT), L-4362 Esch-sur-Alzette, Luxembourg

[2] University of Luxembourg, L- 4365 Esch-sur-Alzette, Luxembourg

[3] Goodyear Innovation Center Luxembourg, L-7750 Colmar-Berg, Luxembourg

[*]Corresponding author: Daniel F. Schmidt (daniel.schmidt@list.lu / (+352) 275 888 4904)

**Characterization of synthesized aminated epoxidized soybean oils**

NMR spectra of modified soybean oils were recorded using an AVANCE III HD Bruker spectrometer (600 MHz, 298 K) using $CDCl_3$ (99.96 %, Sigma Aldrich) as the solvent. The solvent itself was used to provide the reference signal (chloroform, 7.26 ppm). ~2 mg of sample was dissolved in ~500 µL $CDCl_3$ to prepare the sample for NMR experiments. The dissolved sample was then loaded in an NMR tube (Aldrich ColorSpec NMR tube, Sigma Aldrich) and capped properly before placing the tube in the instrument.

$^1$H NMR spectra of samples were collected based on a total number of 16 scans over the range of 0-10 ppm. To enable quantitative measurements, a long relaxation delay (D1) of 40 s was used throughout the study.

Alongside $^1$H NMR spectroscopy, diffusion ordered spectroscopy (DOSY-NMR) was also performed to confirm the success of the grafting reaction. Each DOSY spectrum was collected as an average of 64 scans over the range of 0-10 ppm.

Representative $^1$H NMR signals of the reactants (epoxidized soybean oil and amines) are presented below [δ = chemical shift in ppm (assignment of proton [alphabetic assignment], experimental integration, theoretical integration)], while the $^1$H NMR spectra of the representative samples are shown in Figure S1 – S4 along with their starting materials.

Epoxidized soybean oil (ESO): δ = 5.24-5.30 ppm (**CH** [a], exp 1.00, th 1); 4.10-4.35 ppm (**CH$_2$** [b], exp 4.11, th 4); 2.8-3.2 ppm (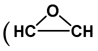

[l], exp 8.60, th 9).

DEA: δ = 2.65-2.70 ppm (N-**CH$_2$** [n′], exp 4.00, th 4); 1.09-1.16 ppm (N-CH$_2$-**CH$_3$** [o′], exp 6.05, th 6). DIPA: δ = 0.98 -1.05 ppm (N-CH-**CH$_3$** [p′], exp 6.00, th 6); 2.84-2.92 ppm (N-**CH** [q′], exp 1.00, th 1). DIBA: δ = 2.36-2.40 ppm (N-**CH$_2$** [r′], exp 4, th 4); 1.67-1.77 ppm (N-CH$_2$-**CH** [s′], exp 2, th 2); 0.83-0.93 ppm (N-CH$_2$-CH-**CH$_3$** [t′], exp 12.25, th 12). bMOEA: δ = 2.65-2.71 ppm (N-C**H$_2$** [u′], exp 4.05, th 4); 3.20-3.27 ppm (O-**CH$_3$** [v′], exp 5.95, th 6); 3.34-3.42 ppm (N-CH$_2$-**CH$_2$** [w′], exp 4.00, th 4).

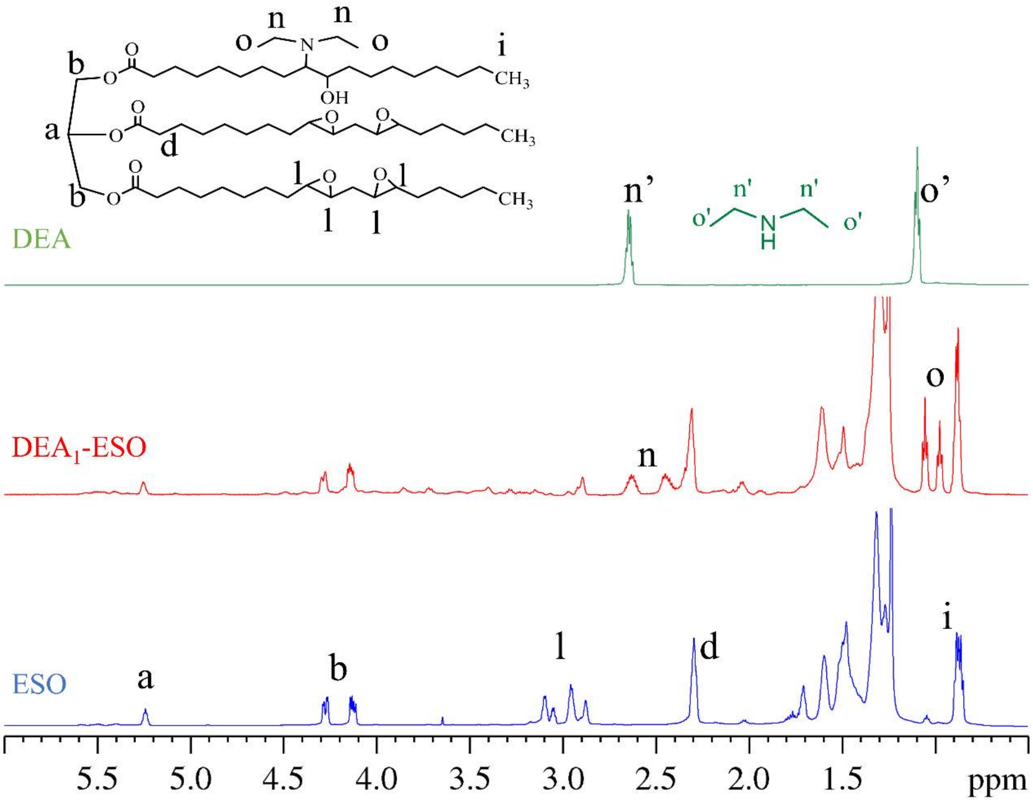


**Figure S1. ¹H NMR spectrum of DEA1-ESO showing the appearance of new signals due to the grafting of the amine, along with the spectra of the starting material (epoxidized soybean oil and DEA)**

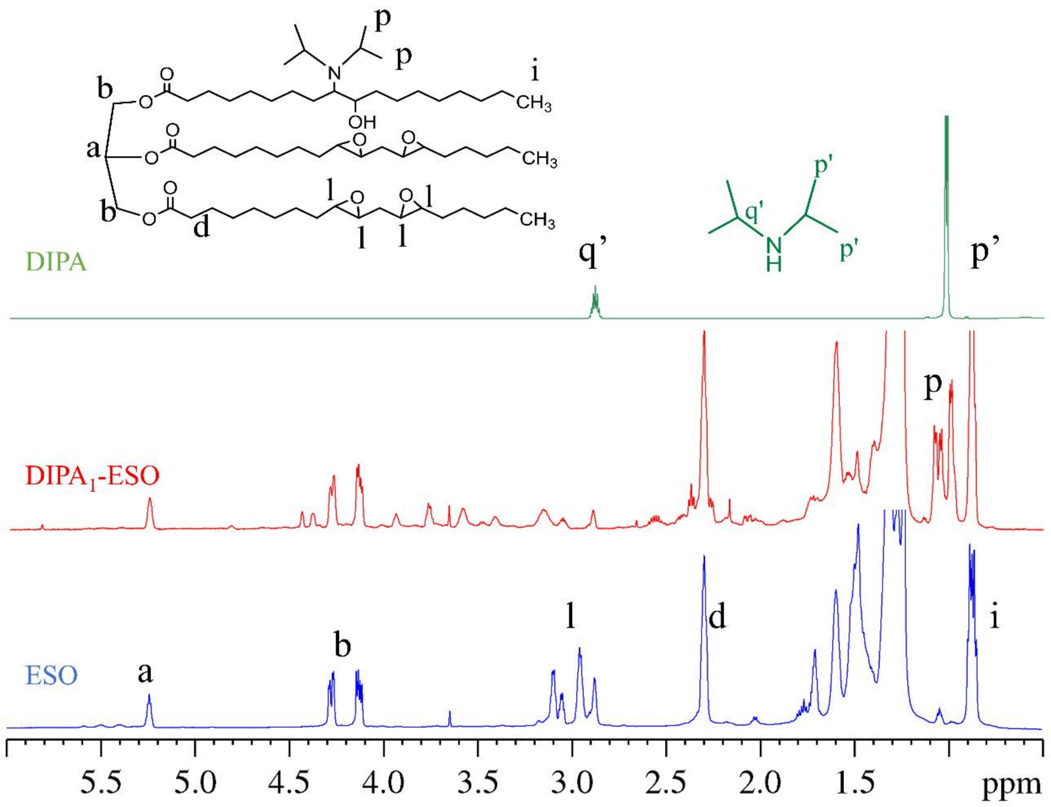


**Figure S2. $^{1}$H NMR spectrum of DIPA₁-ESO showing the appearance of new signals due to the grafting of the amine, along with the spectra of the starting material (epoxidized soybean oil and DIPA)**

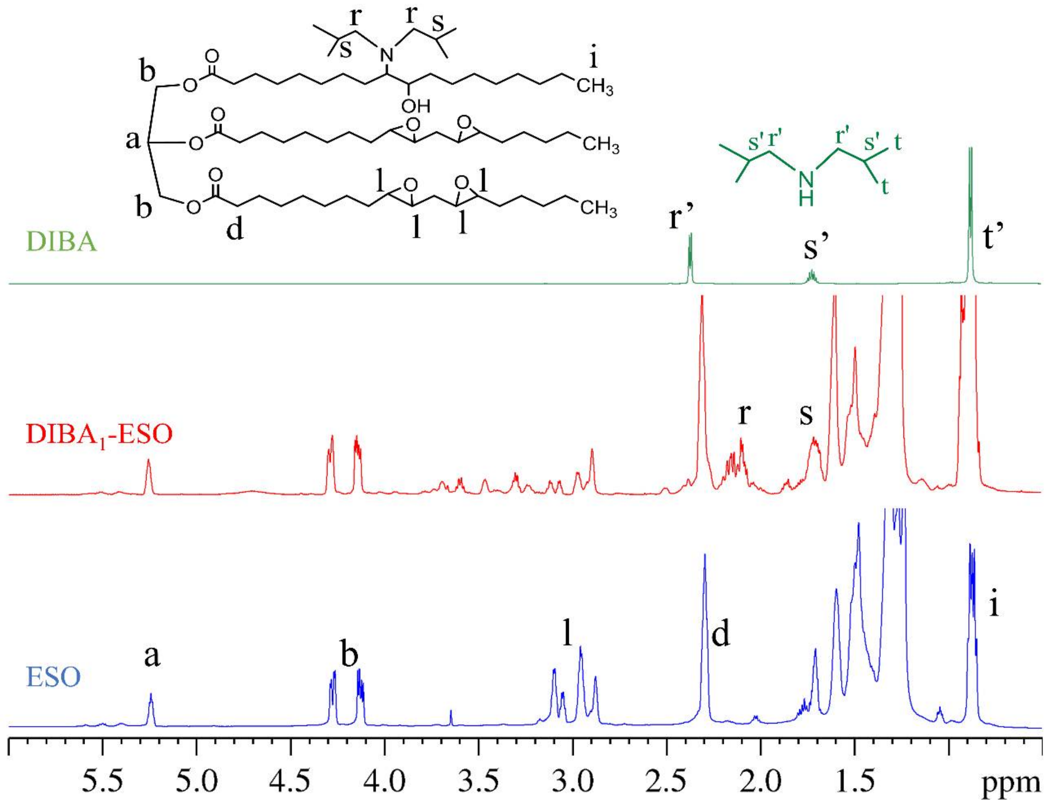


**Figure S3.** $^1$**H NMR spectrum of DIBA**$_1$**-ESO showing the appearance of new signals due to the grafting of the amine, along with the spectra of the starting material (epoxidized soybean oil and DIBA)**

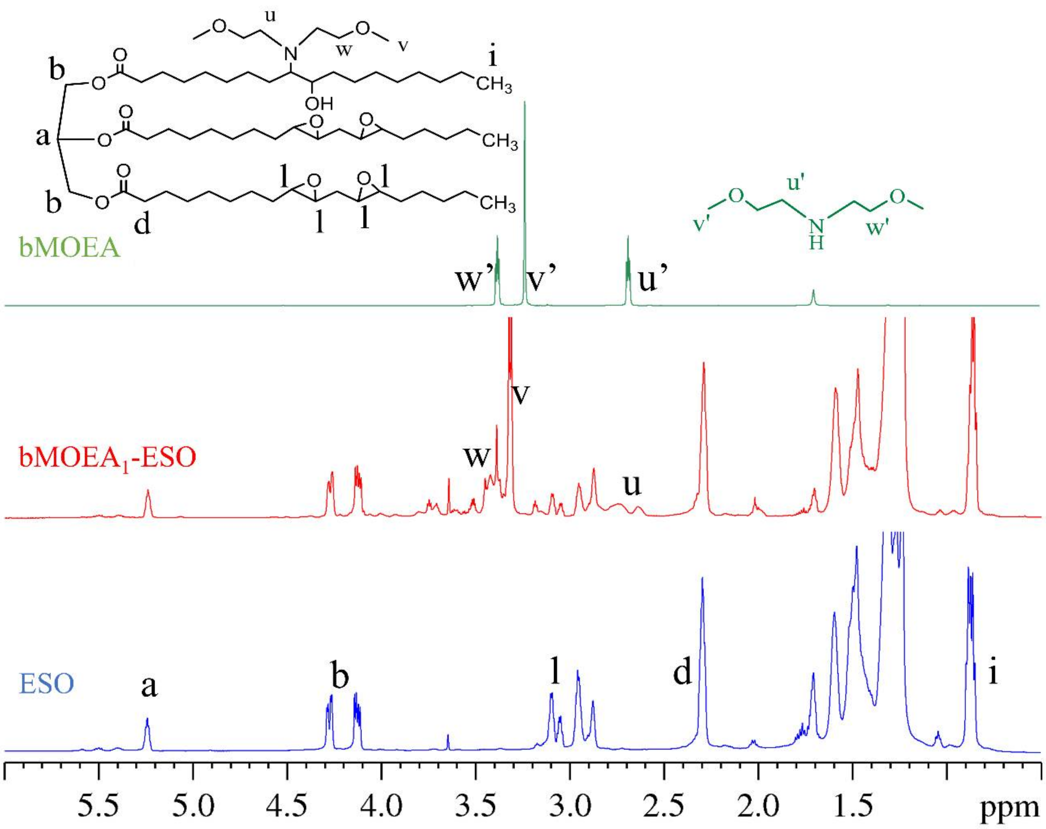


**Figure S4. $^{1}$H NMR spectrum of bMOEA1-ESO showing the appearance of new signals due to the grafting of the amine, along with the spectra of the starting material (epoxidized soybean oil and bMOEA)**

From the $^{1}$H NMR results (Figure S1 - S4), no change in the value of chemical shift for methine (δ = 5.24-5.30 ppm (C**H** [a], exp 1.00)) and methylene hydrogen (δ = 4.10-4.35ppm (C**H**$_2$ [b], exp 4.10) associated with the glycerol part assures the integrity of the triglyceride structure after the amination reaction. Hence, these signals were used as the standard to normalize other peaks of the respective $^{1}$H NMR spectrum. The depletion of epoxy signals (δ = 2.8-3.2 ppm, (HC–O–CH [l])) and the appearance of the new signals corresponding to the characteristic signals of amine in the spectrum of products indicate the presence of grafted amines in the structure of the epoxidized soybean oil. The characteristic new signals of the products providing evidence of the successful amination of epoxidized soybean oil are presented below.

DEA-ESO: δ = 2.42-2.51 and 2.59-2.64 ppm (N-C**H**$_2$ [n]); 0.96-1.03 and 1.07-1.11 (N-CH$_2$-**CH**$_3$ [o]). DIPA-ESO: δ = 0.98-1.13 ppm (N-CH-**CH**$_3$ [p]). DIBA-ESO: δ = 2.08-2.26 ppm (N-**CH**$_2$ [r]); 1.74-1.79 ppm (N-CH$_2$-**CH** [s]). bMOEA-ESO: δ = 2.62-2.92 ppm (N-C**H**$_2$ [u]); 3.31-3.37 ppm (O-C**H**$_3$ [v]); 3.38-3.50 ppm (N-CH$_2$-C**H**$_2$ [w]).

To confirm the amination of epoxidized soybean oil, characterization by DOSY NMR spectroscopy was performed. DOSY NMR works based on the diffusion constant of the molecules, which in turn depends on their hydrodynamic volume . DOSY NMR spectra of the epoxidized oils are shown in Figure S5 along with those for all the reactants. The DOSY NMR spectrum of the product confirms the existence of a single molecular species displaying signals common to both the reacted amine and the epoxidized soybean oil. Taken together, the NMR results imply that amination of the epoxidized soybean oil was successful.

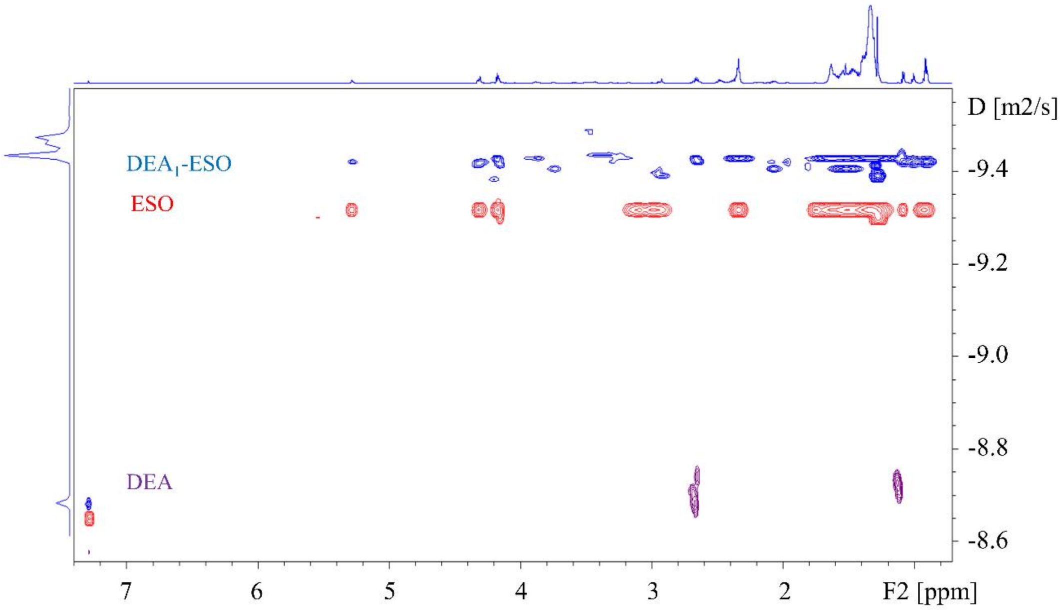


**Figure S5 (a). DOSY NMR of DEA$_1$-ESO along with the parent compound, taken in $CDCl_3$ using an AVANCE III HD Bruker spectrometer operating at 600 MHz**

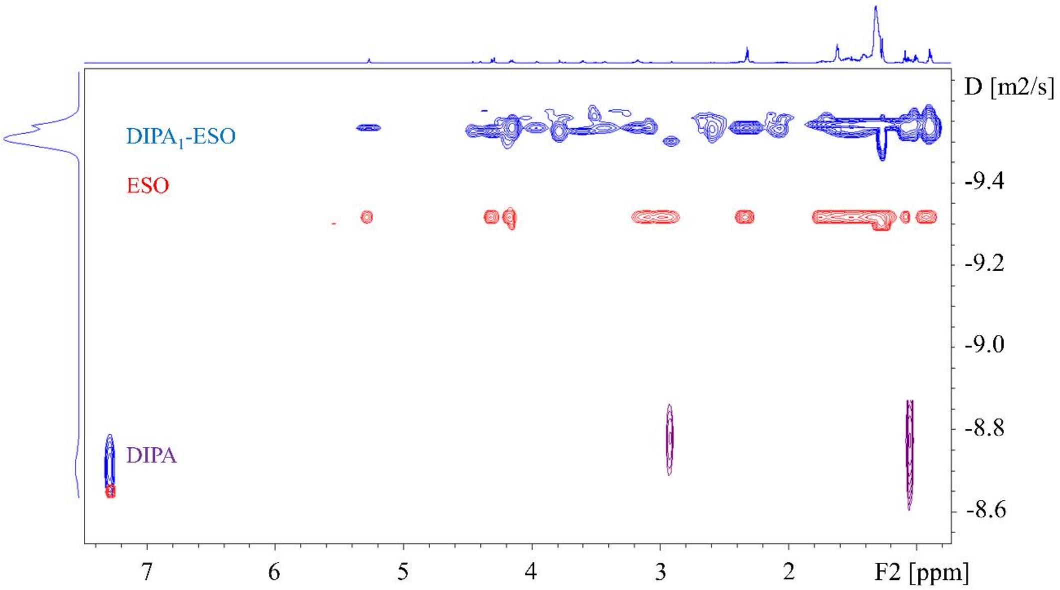


**Figure S5 (b). DOSY NMR of DIPA$_1$-ESO along with the parent compound, taken in $CDCl_3$ using an AVANCE III HD Bruker spectrometer operating at 600 MHz**

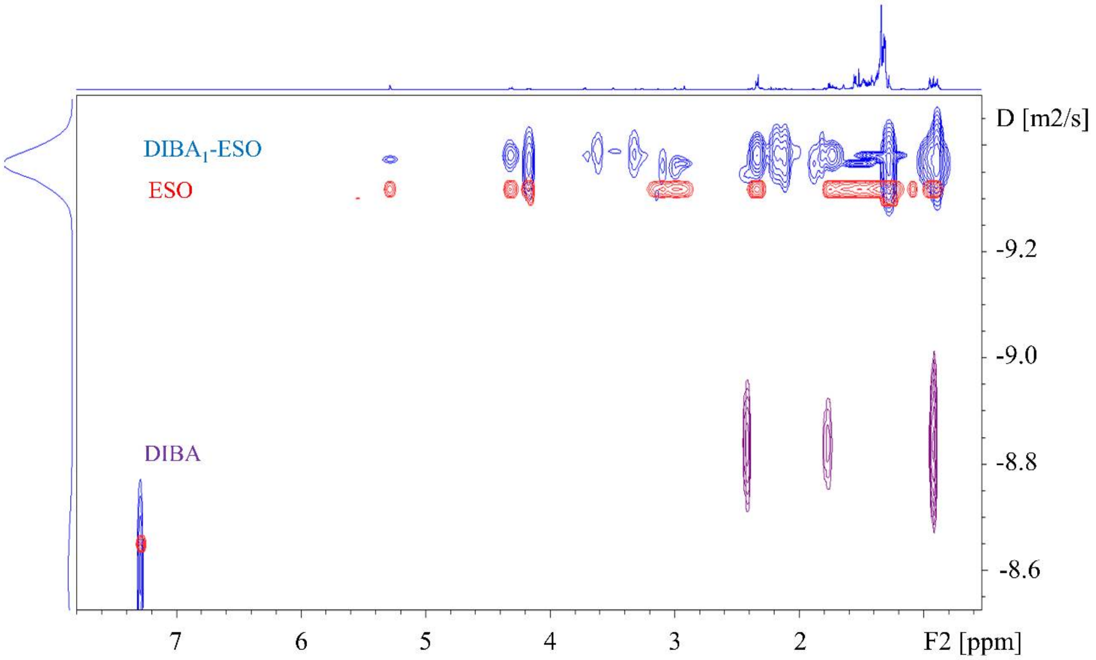


**Figure S5 (c). DOSY NMR of DIBA$_1$-ESO along with the parent compound, taken in $CDCl_3$ using an AVANCE III HD Bruker spectrometer operating at 600 MHz**

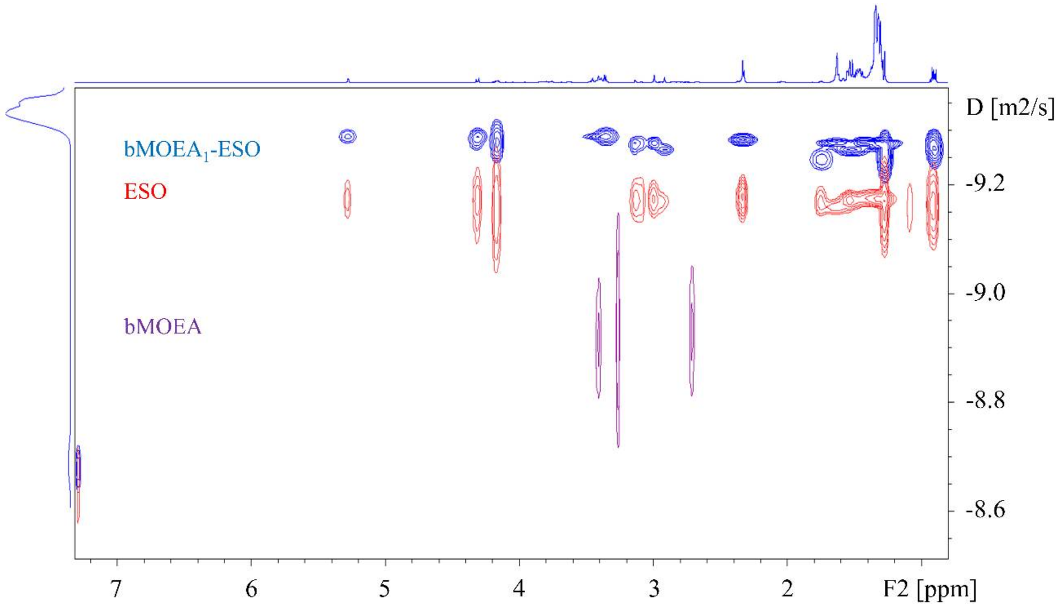


**Figure S5 (d). DOSY NMR of bMOEA$_1$-ESO along with the parent compound, taken in $CDCl_3$ using an AVANCE III HD Bruker spectrometer operating at 600 MHz**

The extent of amination as calculated from the integration of the assigned signals is tabulated in Table S1 along with the respective yields of each reaction. The NMR spectra of the aminated epoxidized soybean oils reported here have been taken from products with ~1 amine per triglyceride; these aminated epoxidized soybean oils have therefore been highlighted in bold in the table to emphasize this point.

Here it is important to mention that the $pK_a$ of the amine did not seem to alter the extent of amination. For instance, diethylamine ($pK_a$ = 11.31) [1] yields aminated epoxidized soybean oil with 1.4 amines per triglyceride after a reaction time of 8 hours, vs. 1.45 amines per triglyceride observed after a reaction time of 8 hours in the case of the equally unhindered bMOEA ($pK_a$ = 8.9) [1]. However, the purification process was observed to affect yield. In particular, the highest yields (80-84 % for DEA-ESO and 89-90 % for DIPA-ESO) were obtained when it was possible to purify the modified oils by vacuum stripping the excess amine. In contrast, column chromatography, necessitated by the higher boiling points of DIBA (138 °C) and bMOEA (170 °C), resulted in lower yields (60-62 % for DIBA-ESO and 40-45 % bMOEA-ESO). The higher polarity of bMOEA-ESO vs. DIBA-ESO may be further depressing the yield given that this could increase retention of the compound on the stationary phase during column chromatography as well.

**Table S1. Dependence of the degree of amination on reaction time for different alkyl amines along with respective yields**

| Amine | Reaction time (hr) | $^1$H NMR peak used* | Amine peak integration | Amines / triglyceride | Reaction yield |
|---|---|---|---|---|---|
| DEA | **4.0#** | δ = 0.96-1.03 & 1.07-1.11 ppm (N-CH$_2$-**CH$_3$**) | **5.85** | **0.99** | 80-84% |
| | 8.0 | | 8.4 | 1.40 | |
| | 12.0 | | 11.89 | 1.98 | |
| | 24.0 | | 11.99 | 2.00 | |
| DIPA | 4.0 | δ = 0.97-1.13 ppm (N-CH-**CH$_3$**) | 6.53 | 0.54 | 62-64% |
| | 8.0 | | 7.13 | 0.59 | |
| | 24.0 | | 8.22 | 0.69 | |
| | **48.0#** | | **10.75** | **0.90** | |
| DIBA | **0.5#** | δ = 2.09-2.25 ppm (N-**CH$_2$**-CH-CH$_3$) | **4.70** | **1.17** | 89-90% |
| | 1.0 | | 6.16 | 1.54 | |
| | 4.0 | | 6.71 | 1.67 | |
| | 24.0 | | 7.89 | 1.97 | |
| bMOEA** | **2#** | δ = [3.31-3.37 ppm, (O-**CH$_3$**), 6H] + [3.38-3.50 ppm, (N-CH$_2$-**CH$_2$**), 4H] | **7.94** | **0.79** | 40-45 % |
| | 8 | | 14.5 | 1.45 | |
| | 24 | | 29.5 | 2.95 | |
| | 36 | | 30.1 | 2.95 | |
| | | **Comments for other amines** | | | |
| bHPA | 4 | The amine is found to attack the triglyceride structure | | | |
| DCHA | 48 | No reaction observed, likely due to steric hindrance | | | |
| Py | 4 | The amine is found to attack the triglyceride structure | | | |
| Pip | 4 | The amine is found to attack the triglyceride structure | | | |
| Az | 4 | The amine is found to attack the triglyceride structure | | | |

*The $^1H$ NMR spectra were normalized to give an integration of 1 in association with the peak associated with the methine hydrogen of the triglyceride (δ = 5.25 ppm; C**H**), which was used as a reference.
#Denotes reaction times required to produce aminated epoxidized soybean oils with an amine content of ~1 amine per triglyceride ($A_1$-ESO, "A" refers to the type of amine).
**As the peaks at δ = (3.31-3.37 ppm; (O-CH3); 6H) and δ = (3.38-3.50 ppm; (N-CH2-CH2); 4H) both correspond to bMOEA and overlap one another, the sum of their integrations (corresponding to 10 H) is considered when estimating amines/triglyceride.

FTIR studies of modified soybean oils were performed in attenuated total reflectance (ATR) mode using a Bruker Tensor 27 instrument with an integrated diamond ATR crystal. Background scans were performed before each experiment. A small amount of sample (0.025-0.050 g) was then placed on the crystal, and spectra were collected based on a total of 32 scans at a resolution of 4 $cm^{-1}$ over the range of 4000 to 600 $cm^{-1}$. After each experiment, the crystal was cleaned using ethanol (95 %, Acros Organics) and soft tissue paper.

The FTIR spectra of the same representative aminated epoxidized oil samples that formed the basis for the NMR section (i.e. with ~1 amine per triglyceride) is shown in Figure S6 (a-d). From the spectra, the reduced intensity of the epoxy peak and the appearance of the hydroxyl peak support the conclusions reached via NMR analyses concerning the reaction of the epoxy groups. The unaltered peak position and the peak height of the carbonyl group and the absence of an amide peak further confirms the integrity of the triglyceride structure and the lack of any amidation of the triglyceride esters. Overall, the FTIR analysis supports the presence of a triglyceride structure containing ring-opened epoxy groups, consistent with the NMR analyses.

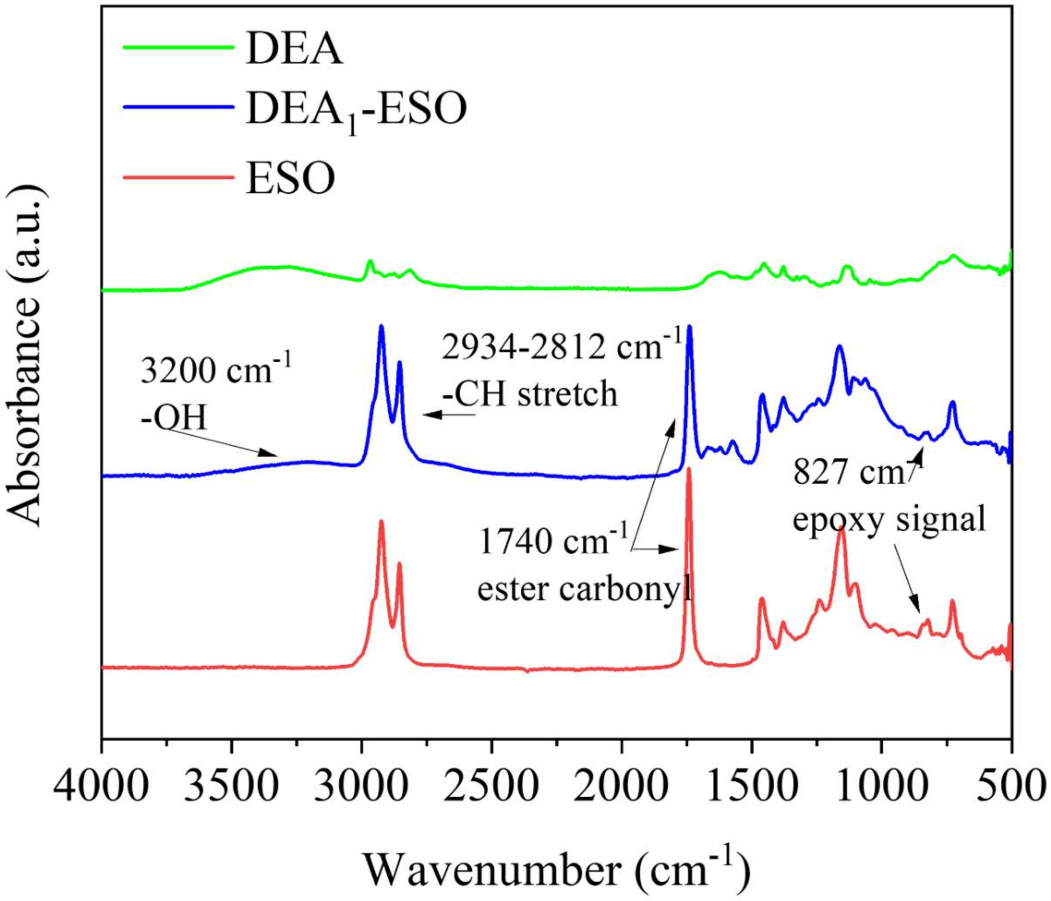


**Figure S6 (a). FTIR of $DEA_1$-ESO along with the starting material performed in ATR mode**

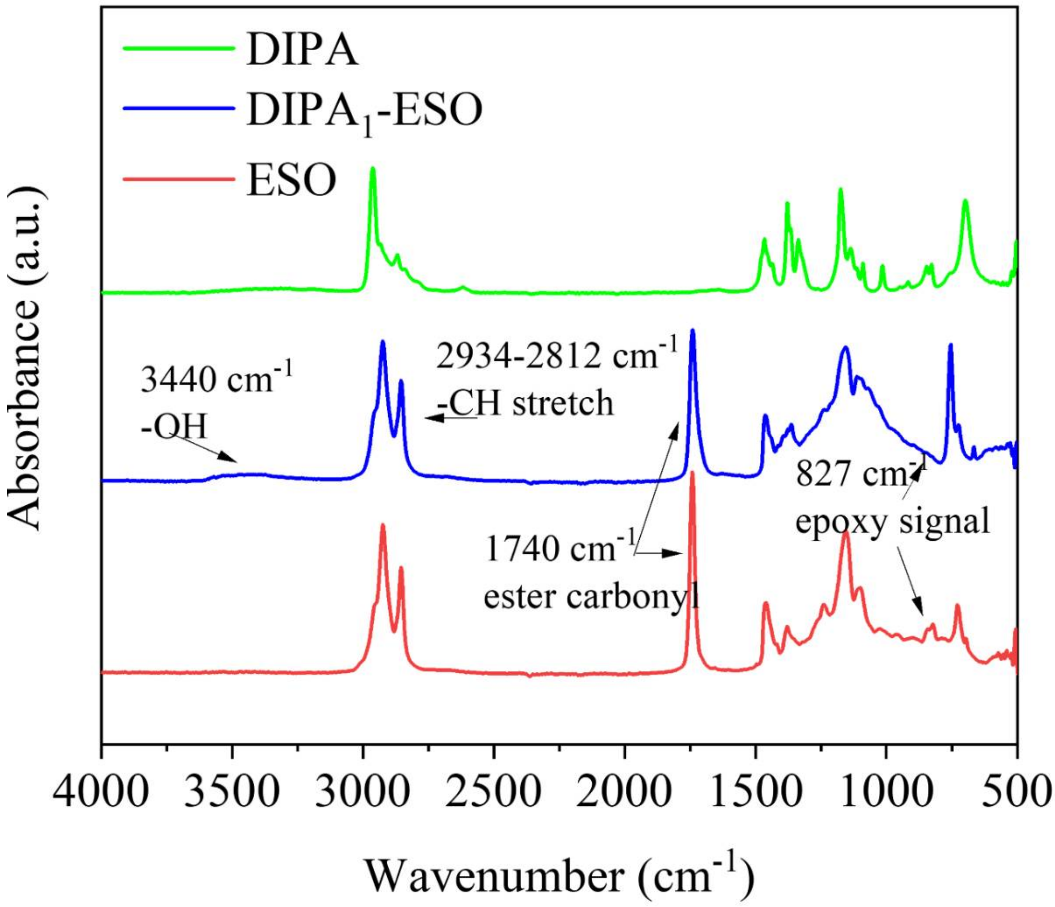


**Figure S6 (b). FTIR of DIPA₁-ESO along with the starting material performed in ATR mode**

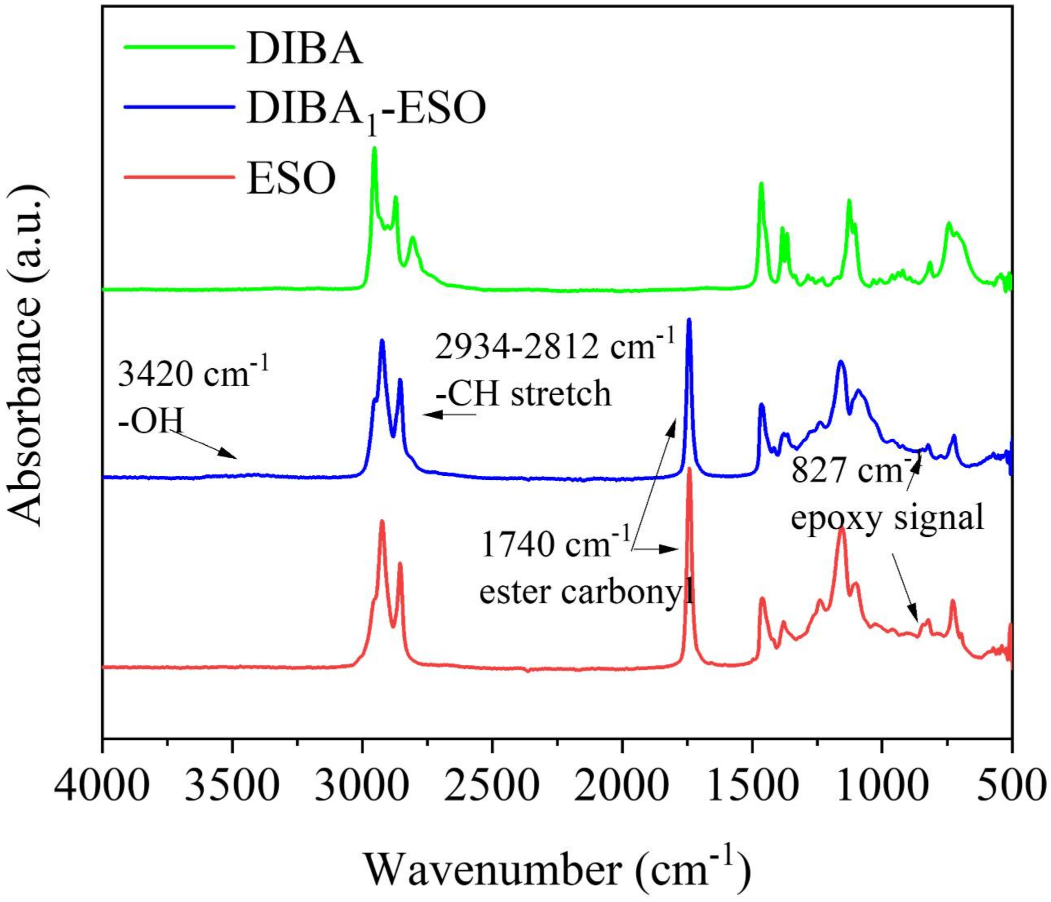


**Figure S6 (c). FTIR of DIBA$_1$-ESO along with the starting material performed in ATR mode**

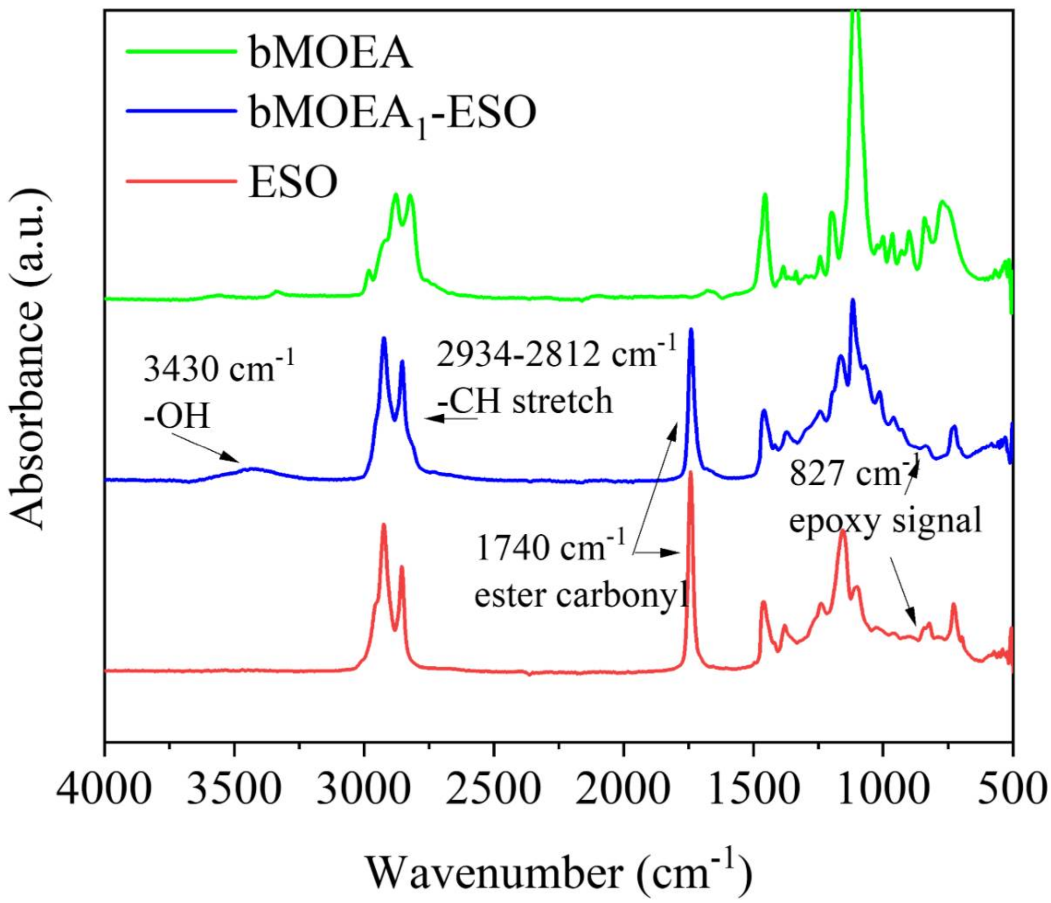


**Figure S6 (d). FTIR of bMOEA$_1$-ESO along with the starting material performed in ATR mode**

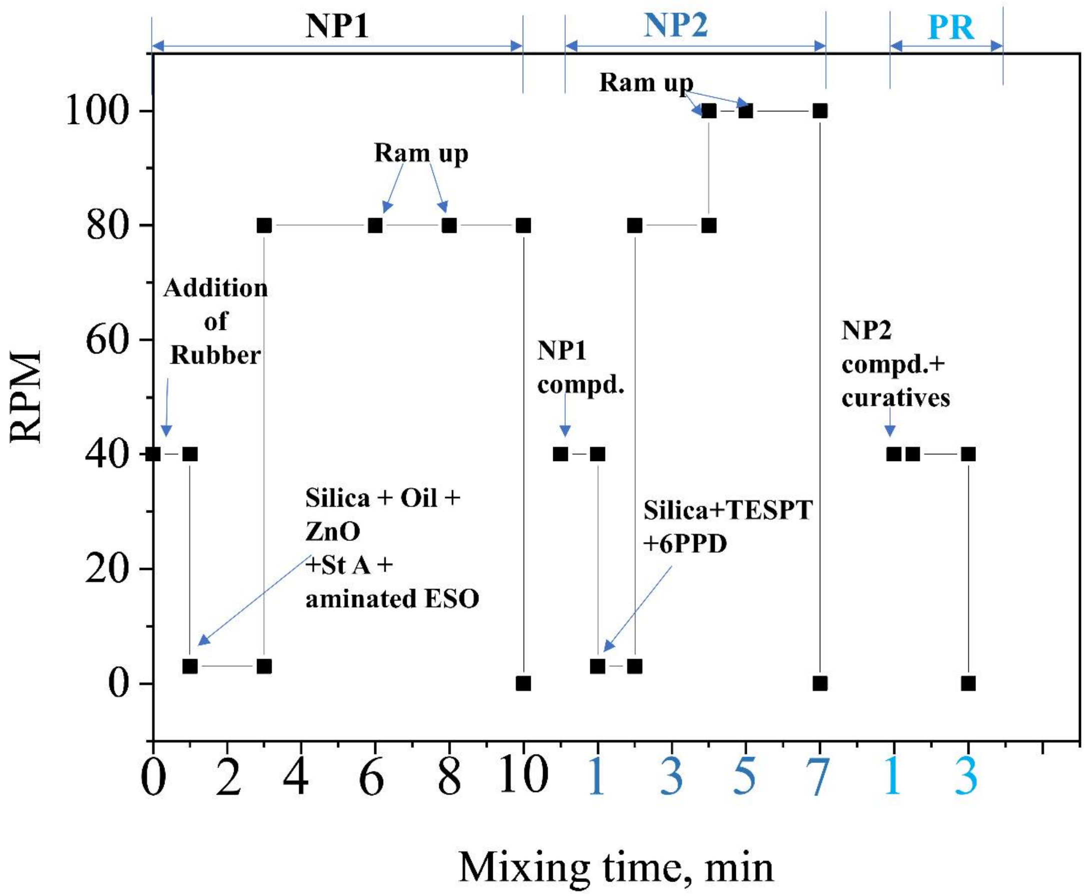


**Figure S7. General mixing plan for the rubber compounds using a small-scale internal mixer (free volume 85 cc, Haake) pre-heated to 80 °C at the beginning of the NP_1 and NP_2 steps and 60 °C at the beginning of the PR step, respectively.**

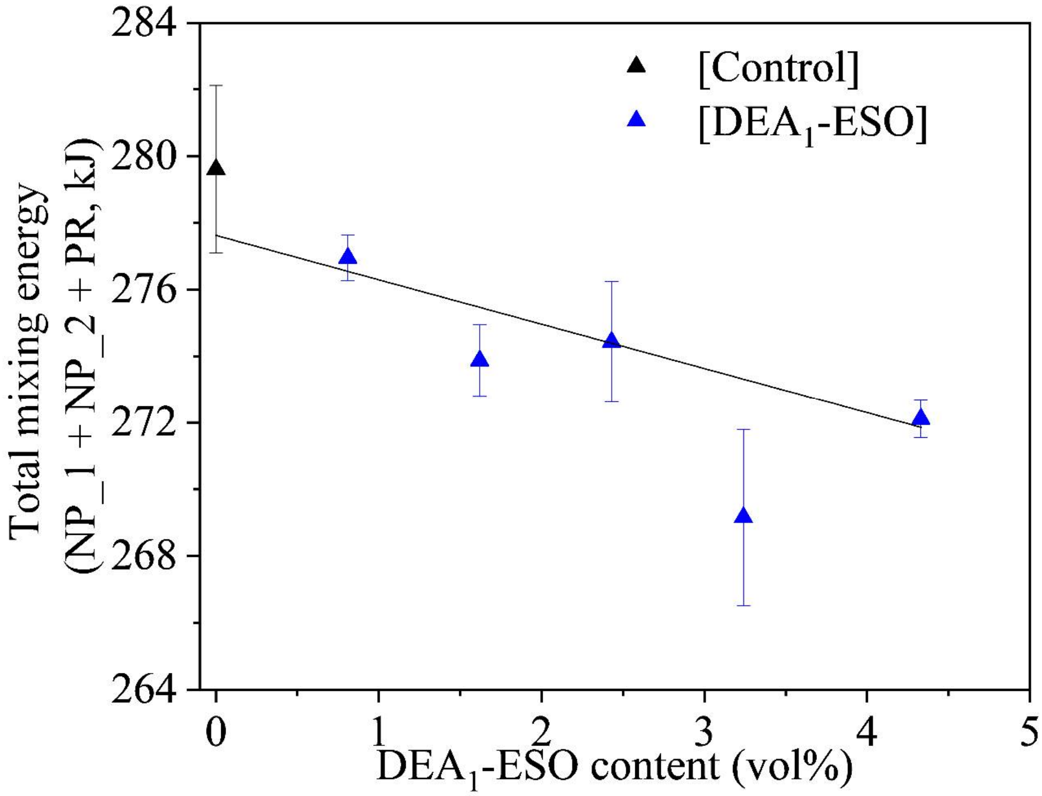


**Figure S8. The cumulative mixing energies for the whole course of mixing of the silica-filled rubber compounds vs. volume fraction of aminated epoxidized soybean oil ($DEA_1$-ESO) in the rubber formulation, including the control**

**Table S2. Mixing energy for silica-filled SBR samples as a function of degree of amination of aminated epoxidized soybean oil (DEA-ESO)**

| Name of oil | Number of amine groups per triglyceride | Content (phr) | | Mixing energy (kJ) |
|---|---|---|---|---|
| | | TDAE oil | DEA-ESO | |
| TDAE oil | -- | 26 | 0 | 155 (± 2) |
| $DEA_1$-ESO | 0.99 (± 0.05) | 21.5 | 4.5 | 143 (± 1) |
| $DEA_{1.4}$-ESO | 1.40 (± 0.05) | 21.5 | 4.5 | 144 (± 2) |
| $DEA_2$-ESO | 1.98 (± 0.05) | 21.5 | 4.5 | 143 (± 2) |

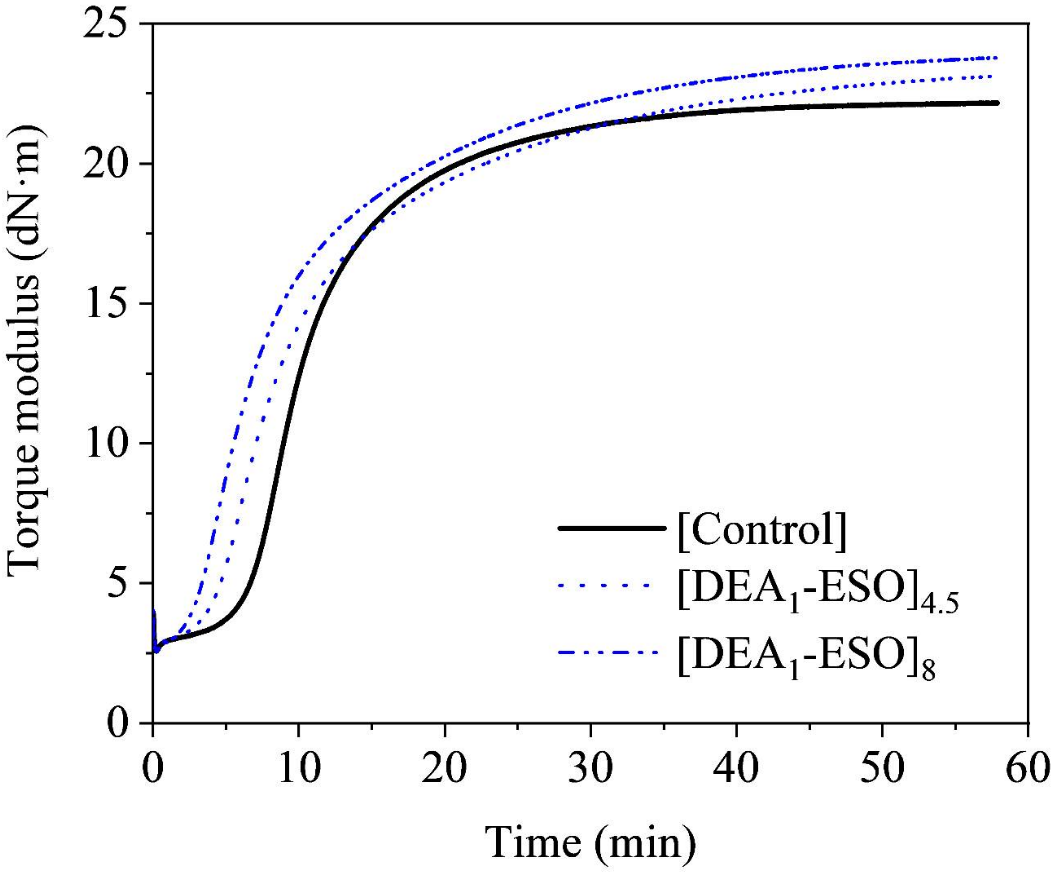


**Figure S9. Cure curves (from MDR) for silica-filled SBR samples as a function of aminated epoxidized soybean oils (DEA1-ESO) content**

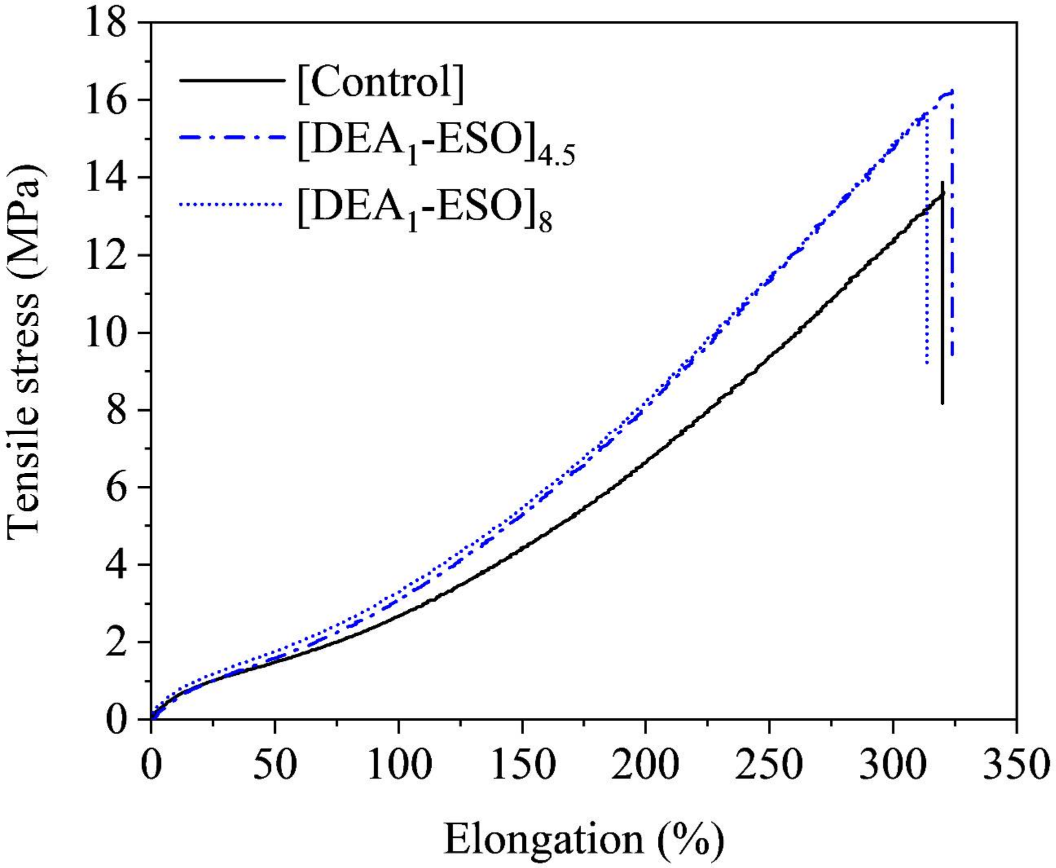


**Figure S10. Quasi-static mechanical properties of silica-filled SBR samples as a function of aminated epoxidized soybean oil (DEA$_1$-ESO) content; stress vs strain curves showing the average measured tensile strength values are chosen for comparison**

Various morphological parameters are derived from the μCT analyses. Equivalent diameter ($D_{eq}$) corresponds to the diameter of a sphere with the same volume as the object being analyzed. These values are defined as follows:

$$D_{eq} = \sqrt[3]{\frac{6V}{\pi}}; \tag{S1}$$

$$G_1 = \frac{\sqrt{n(n-1)}}{n-2}\left[\frac{\frac{1}{n}\sum_{i=1}^{n}(x_i-\bar{x})^3}{\left(\frac{1}{n}\sum_{i=1}^{n}(x_i-\bar{x})^2\right)^{3/2}}\right] \tag{S2}$$

where $V$ is the volume of the object, $V_{total}$ is the total analyzed volume, $n$ is the number of heterogeneities to be analyzed (the sample size), $x_i$ represents the set of all sampled equivalent diameter values, and $\bar{x}$ is the sample mean equivalent diameter.

**References**

1. Lobo, S.T.; Robertson, R.E. Thermodynamics of basic ionization of some aminoethers in water. *Canadian Journal of Chemistry* **1976**, *54*, 3600-3606.
